# Loss of cortactin causes endothelial barrier dysfunction via disturbed adrenomedullin secretion and actomyosin contractility

**DOI:** 10.1038/srep29003

**Published:** 2016-06-30

**Authors:** Alexander García Ponce, Alí F. Citalán Madrid, Hilda Vargas Robles, Sandra Chánez Paredes, Porfirio Nava, Abigail Betanzos, Alexander Zarbock, Klemens Rottner, Dietmar Vestweber, Michael Schnoor

**Affiliations:** 1 Department for Molecular Biomedicine, Center of Research and Advanced Studies (CINVESTAV-IPN), 07360 Mexico-City, Mexico; 2 Department for Physiology, Biophysics and Neurosciences, Center of Research and Advanced Studies (CINVESTAV-IPN), 07360 Mexico-City, Mexico; 3 Department for Infectomics and Molecular Pathogenesis, Center of Research and Advanced Studies (CINVESTAV-IPN), 07360 Mexico-City, Mexico; 4 Department of Anesthesiology, Intensive Care and Pain Medicine, University Clinic of Münster, 48149 Münster, Germany; 5 Division of Molecular Cell Biology, Zoological Institute, TU Braunschweig, 38106 Braunschweig, Germany; 6 Department of Cell Biology, Helmholtz Centre for Infection Research, 38124 Braunschweig, Germany; 7 Department for Vascular Cell Biology, Max-Planck-Institute for Molecular Biomedicine, 48149 Münster, Germany

## Abstract

Changes in vascular permeability occur during inflammation and the actin cytoskeleton plays a crucial role in regulating endothelial cell contacts and permeability. We demonstrated recently that the actin-binding protein cortactin regulates vascular permeability via Rap1. However, it is unknown if the actin cytoskeleton contributes to increased vascular permeability without cortactin. As we consistently observed more actin fibres in cortactin-depleted endothelial cells, we hypothesised that cortactin depletion results in increased stress fibre contractility and endothelial barrier destabilisation. Analysing the contractile machinery, we found increased ROCK1 protein levels in cortactin-depleted endothelium. Concomitantly, myosin light chain phosphorylation was increased while cofilin, mDia and ERM were unaffected. Secretion of the barrier-stabilising hormone adrenomedullin, which activates Rap1 and counteracts actomyosin contractility, was reduced in plasma from cortactin-deficient mice and in supernatants of cortactin-depleted endothelium. Importantly, adrenomedullin administration and ROCK1 inhibition reduced actomyosin contractility and rescued the effect on permeability provoked by cortactin deficiency *in vitro* and *in vivo*. Our data suggest a new role for cortactin in controlling actomyosin contractility with consequences for endothelial barrier integrity.

Endothelial cells provide a selective barrier within the vasculature to separate the blood from tissues. During inflammation, pro-inflammatory cytokines activate endothelial cells by induction of adhesion molecule expression and loosening of integral membrane structures called tight and adherens junctions that form the inter-endothelial cell contacts. Such activation leads to leukocyte transmigration and leakage of blood components into tissues^1^,^2^,^3^. The maintenance of the endothelium as a semi-permeable barrier is particularly important for the passage of macromolecules and fluid between the blood and interstitial space. If not controlled properly, excessive leakage may result in tissue inflammation and inflammatory diseases such as acute respiratory distress syndrome, arthritis or sepsis^4^,^5^.

An important mechanism that regulates endothelial junctions and thus vascular permeability is the remodeling of the actin cytoskeleton into either a stabilising cortical actin ring or destabilising contractile stress fibres. Actin is one of the most abundant and highly conserved proteins in mammalian cells^6^. Under physiological conditions, actin exists as globular actin which assembles into organized filaments that, together with microtubules, intermediate filaments and septins, constitute the cytoskeleton. Actin filaments support many cellular functions such as movement of organelles, adhesion, migration, endocytosis and formation of lamellipodia and filopodia among others^7^,^8^. At cell-cell contacts, actin is linked indirectly via adaptor proteins such as catenins and zonula occludens (ZO) to transmembrane adhesion molecules present at adherens junctions (AJ) (e.g. VE-cadherin) and tight junctions (TJ) (e.g. occludin)^9^,^10^. Actin filaments anchor endothelial cells to each other and to the extracellular matrix, thus maintaining endothelial monolayer integrity. By contrast, specialized actin filament structures such as stress fibres, that form under inflammatory conditions, act in concert with myosin and α-actinin to induce actomyosin contractility that generate pulling forces and destabilise endothelial contacts^11^,^12^.

We recently showed that the actin-binding protein cortactin is required for the proper control of endothelial barrier integrity *in vivo*^13^. Cortactin is an F-actin binding protein of 80/85 kDa encoded by the EMS1 (Excess Microsporocytes 1) gene, located on chromosome 11, in the 11q13 region, frequently amplified in different cancer types^14^,^15^. Cortactin is ubiquitously expressed except for most hematopoietic cells and contains the following key domains: an N-terminal acidic domain that can bind to the Arp2/3 complex, an F-actin binding domain, a proline-rich region containing serine and tyrosine phosphorylation sites and a SH3 domain that mediates interaction with several proteins involved in actin polymerization (N-WASP), cell–cell adhesion (ZO-1) and membrane dynamics (dynamin)^16^,^17^,^18^. Cortactin can interact with myosin-light chain kinase (MLCK) in endothelial cells^19^,^20^, but a direct contribution of cortactin to MLC phosphorylation and actomyosin contractility has not been investigated. Instead, it has been shown that cortactin translocates to endothelial cell contacts in response to shear-stress where it may contribute to actin remodeling^21^.

Cortactin-deficient murine lung endothelial cells (MLEC) and cortactin-depleted human umbilical vein endothelial cells (HUVEC) show higher permeability than wild type cells due to reduced levels of active Rap1 13. Rap1 is known to stabilise endothelial junctions, whereas another small GTPase, RhoA, stimulates actomyosin contractility by MLC phosphorylation via its effector Rho-kinase 1 (ROCK1) leading to increased endothelial permeability^22^,^23^,^24^. Of note, active Rap1 can inhibit RhoA^25^. Thus, it is tempting to speculate that defective Rap1 activation in the absence of cortactin affects the RhoA/ROCK axis to increase actomyosin contractility and endothelial permeability.

Besides its known role as actin filament and Arp2/3 complex interactor, we now provide evidence for the first time that cortactin also regulates ROCK1-mediated actomyosin contractility in endothelial cells. Furthermore, we found that loss of cortactin causes decreased adrenomedullin (ADM) secretion. ADM is known to stabilise the endothelial barrier by inducing cAMP production. Given that cAMP activates Rap1, stabilises cortical actin and reduces stress fibres, these findings likely explain the lower levels of active Rap1 and increased actomyosin contractility in cortactin-deficient endothelial cells. Thus, these new data combined with our previous observations provide a mechanistic feedback-loop explaining endothelial barrier dysfunction in the absence of cortactin.

## Results

### Loss of cortactin leads to altered actin cytoskeleton morphology

**Figure 1:**
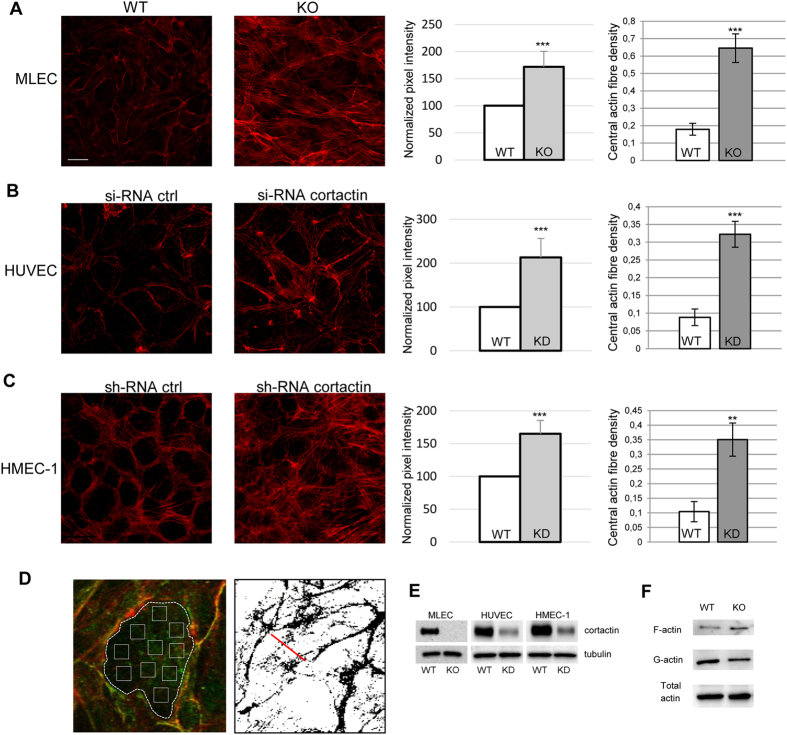
Cortactin depletion results in altered actin morphology in endothelial cells. Cortactin-deficient murine lung endothelial cells (MLEC, **A**), cortactin-depleted HUVEC (**B**) and HMEC-1 (**C**) were analysed for actin morphology by fluorescence using Alexa568-phalloidin. Images are representative of three independent experiments. Bar = 20 μm. Pixel intensities (graphs on the right) were quantified using ImageJ software. Graphs show means +/−SDM of three independent cultivations. ***p < 0.001. (**D**) Left: Image illustrating the method of actin fibre quantification as depicted in the left bar charts shown in Fig. 1A–C. Actin staining in red; VE-cadherin staining in green. Right: Illustration of assessment of central actin fibre density^28^, results of which are shown in the respective right bar charts of Fig. 1A–C. (**E**) Verification of cortactin depletion or deficiency by Western blot. (**F**) Analysis of G- vs F-actin levels, and total actin content in lung lysates of WT and cortactin KO mice. Representative blots of three independent experiments are shown.

We recently demonstrated that loss of cortactin causes endothelial barrier dysfunction due to decreased levels of active Rap1 *in vitro* and *in vivo*^13^. Additionally, we and others have observed more actin fibres in cortactin-deficient and cortactin-depleted endothelial cells^13^,^26^, but the mechanisms how cortactin deficiency affects actin dynamics in endothelial cells remain elusive. Given the well-known functions of cortactin in actin remodeling and the importance of actin and actin-binding proteins for endothelial barrier homeostasis^9^,^27^, we asked if altered actin dynamics could contribute to the increased vascular permeability. First, we analysed the structure of the actin cytoskeleton in the absence of cortactin in different types of endothelia. As can be seen in Fig. 1A, primary cortactin-deficient murine lung endothelial cells (MLEC) showed increased actin fibres crossing the cell body. Quantifying these actin structures by determining the pixel intensity of these fibres only (i.e. explicitly excluding cortical actin as depicted in Fig. 1D), we found a significant increase in such actin fibres (Fig. 1A, middle panel). Then we asked if this is true also for other endothelial cell types. HUVEC, transiently depleted of cortactin by siRNA (Fig. 1B), and human microvascular endothelial cells (HMEC-1), stably cortactin-depleted by shRNA-expressing lentivirus (Fig. 1C), both showed a significant increase in these actin fibres to an extent comparable to primary, cortactin-deficient MLEC. Additionally, we applied a stress fibre density quantification method as depicted in Fig. 1D described by Peacock and colleagues^28^. These measurements invariably revealed a significant increase in actin fibres crossing the cell centers of each cell type (Fig. 1A–C; right panels). Control blots for cortactin depletion and deficiency, respectively, are shown in Fig. 1E. Using a commercial F-actin/G-actin extraction kit, we could confirm an increase in filamentous actin with no changes in total actin (Fig. 1F). Concomitantly, the amount of G-actin was reduced without cortactin. Quantification of three independent experiments revealed a statistically significant shift of the F/G-actin ratios from 0.738+/−0.075 in the WT to 1.293+/−0.062 in KO. These data clearly show that formation of F-actin is favored in endothelial cells that lack cortactin.

### Loss of cortactin leads to increased ROCK-1 levels

**Figure 2:**
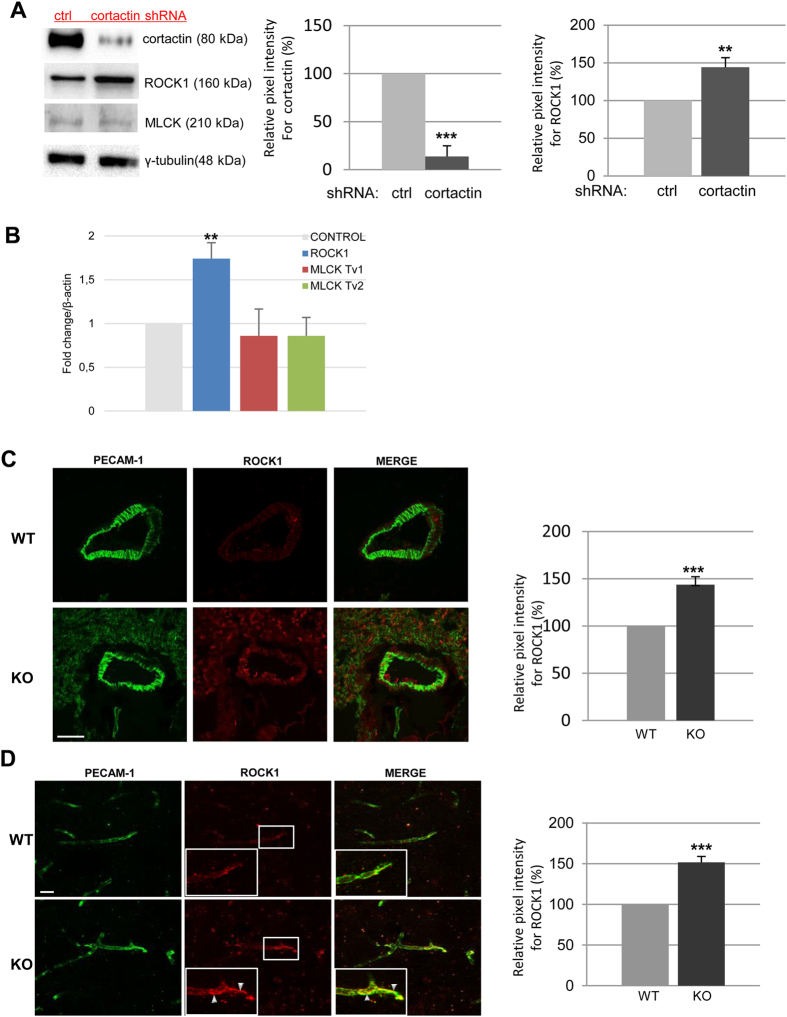
Absence of cortactin causes increased ROCK1 protein levels. Cortactin-depleted HMEC-1 and scrambled control cells were grown to confluency and lysed for either protein or RNA extraction. (**A**) Proteins were analysed by western blot for cortactin, MLCK, and ROCK1 expression. γ-tubulin served as loading control. Left graph shows quantification of cortactin expression, and right graph shows quantification of ROCK1 expression of three independent experiments each. **p < 0.01; ***p < 0.001 (**B**) cDNAs were analysed by real-time RT-PCR for expression of ROCK1 and MLCK (transcript variants (Tv) 1 and 2). Data were normalized to β-actin as house-keeping gene, and WT controls were set to 1 for each amplicon. Cryotissue sections from lungs (**C**) and brains (**D**) were analysed by immunofluorescence using anti-PECAM-1 and anti-ROCK1 antibodies. White squares indicate zoomed areas of interest in brain vessels (**D**) where colocalization of PECAM-1 and ROCK-1 can be observed (arrowheads). Bars, 20 μm (brains) and 10 μm (lungs). Pixel intensities for ROCK1 (graphs on the right) were quantified using ImageJ software. Graphs show means +/−SDM of three (lungs) and four (brains) independent tissue preparations. ***p < 0.001.

Next, we wanted to know if the observed actin structures represented contractile actomyosin stress fibres which are known to destabilise endothelial cell contacts due to the pulling forces they generate^29^,^30^. To this end, we first analysed the expression of the kinases known to induce actomyosin contractility by phosphorylating myosin light chain (MLC), ROCK1 and MLC kinase (MLCK), in stably cortactin-depleted HMEC-1 cells. This stable endothelial cell line showed a cortactin silencing efficiency of 86% (+/−5.7%) (Fig. 2A, left and middle panel). Interestingly, we found that the protein expression levels of ROCK1 were increased by 45.6% (+/−9.3%) (Fig. 2A, left and right panel) in cortactin-depleted cells while levels of MLCK appeared slightly reduced by 15.85+/−5.67% (left panel, quantification not shown), although this was not statistically significant. To analyse if increased ROCK1 protein levels were a consequence of increased transcription of ROCK1 mRNA, we performed real-time RT-PCR. As shown in Fig. 2B, ROCK1 mRNA levels showed a significant increase (1.74+/−0.18 fold) in expression when normalised to the expression levels of the house-keeping gene β-actin. MLCK expression was not significantly changed.

In order to confirm a physiological relevance of these findings, we analysed ROCK1 levels and localization in lung (Fig. 2C) and brain (Fig. 2D) cryosections obtained from C57Bl/6J WT and cortactin KO mice. To this end, IF stainings for ROCK1 in combination with PECAM-1 as a blood vessel marker were performed. We observed a statistically significant increase of 44.6% (+/−7.9%) in ROCK1 expression in blood vessels of lungs (Fig. 2C) and of 51.6% (+/−7.1%) in blood vessels of brains (Fig. 2D). Control stainings using only secondary antibodies did not yield considerable signals (data not shown). Interestingly, ROCK1 partly co-localized with PECAM-1 at endothelial cell contacts only in brain sections of the KO mice (zoomed areas in white squares). Our *in vitro* and *ex vivo* data demonstrate that loss of cortactin increases ROCK1 protein levels in endothelial cells.

### Cortactin depletion increases MLC phosphorylation but does not affect expression or activation of other proteins involved in actin stress fibre formation

**Figure 3:**
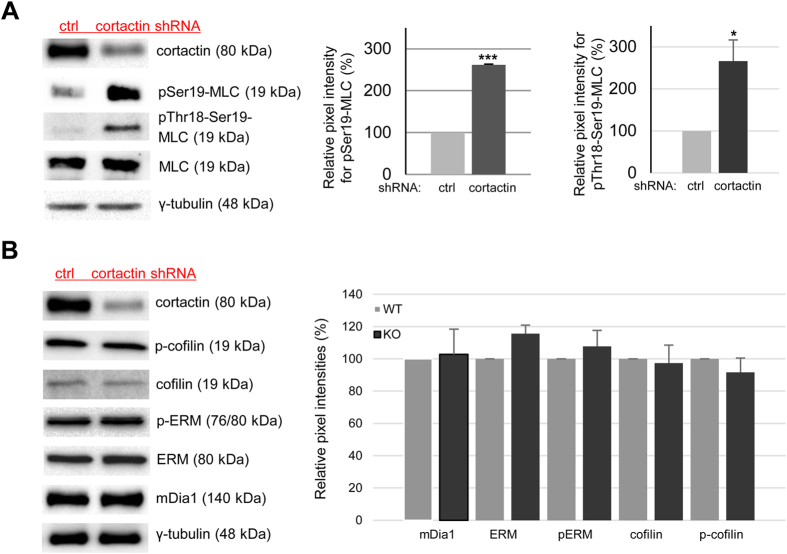
Cortactin depletion leads to increased MLC phosphorylation whereas levels of cofilin, mDia1 and ERM are unaffected. (**A**) Loss of cortactin causes a strong increase of MLC phosphorylation at both Thr18 and Ser19 in cortactin-depleted HMEC-1 while total MLC levels remain unaffected. Graphs show quantification of MLC phosphorylation (Ser19, middle panel; Thr18-Ser19, right panel) of three independent experiments. ***p < 0.001 (**B**) Western blots showing no differences in production and phosphorylation of other proteins implicated in stress fibre formation such as ERM, cofilin or mDia1. Representative blots of three independent experiments are shown.

ROCK1 induces actomyosin contractility by direct phosphorylation of MLC at threonine-18 and serine-19 31,32. Thus, we speculated that the increase in ROCK1 protein levels in cortactin-deficient cells could lead to increased MLC phosphorylation and thus increased actin stress fibre contractility. As shown in Fig. 3A, we observed a 168+/−6.4% increase in MLC phosphorylation at serine-19 in cortactin-depleted endothelial cells. Using an antibody specifically recognizing MLC only when dually phosphorylated at Thr-18 and Ser-19, we detected a similar increase of 166+/−43.8% (Fig. 3A). Representative blots document unchanged total MLC levels and the extent of cortactin depletion; γ-tubulin was used as loading control (Fig. 3A). Next, we wanted to know if loss of cortactin affected mDia1 or cofilin as potential regulators of actin fibre formation and turnover. As shown in Fig. 3B, protein levels of mDia1 and cofilin as well as phosphorylation levels of cofilin were not changed by cortactin depletion. Moreover, levels of ezrin, radixin and moesin (ERM) and phosphorylated ERM, known to play a role in stress fibre formation^33^,^34^, were not altered by the loss of cortactin. We conclude that cortactin plays a role in the regulation of actomyosin contractility in endothelial cells by affecting ROCK1-mediated MLC phosphorylation.

### Cortactin is required for proper adrenomedullin secretion

**Figure 4:**
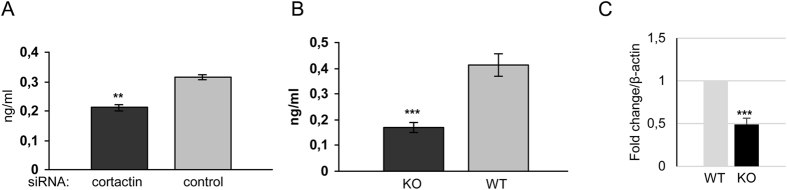
Adrenomedullin is secreted to a lesser extent without cortactin. (**A**) Confluent HUVEC monolayers were cultured for 3 days in fresh medium before collection of supernatants and analysis of ADM concentrations by ELISA. (**B**) Blood from WT and cortactin KO mice was extracted from the retro-orbital plexus. Plasma was analysed by ELISA to determine ADM concentrations. Graphs show means +/−SDM of three independent cell cultivations or 6 mice, respectively. (**C**) cDNA was analysed by real-time RT-PCR for expression of ADM. Data are normalized to β-actin as house-keeping gene.**p < 0.01; ***p < 0.001.

Previous reports have demonstrated that cAMP-induced activation of the EPAC-Rap1 pathway counteracts vascular hyperpermeability^35^,^36^. *In vivo*, the regulatory peptide hormone adrenomedullin (ADM), which is produced by several cell types including endothelial cells, enhances endothelial barrier function by inducing cAMP production, Rap1 activation and VE-cadherin-dependent cell-cell adhesion^37^,^38^,^39^,^40^. Thus, we hypothesized that decreased Rap1 activity^13^ and increased actomyosin contractility in the absence of cortactin may be due to altered adrenomedullin production. In order to test this hypothesis, supernatants of confluent control and cortactin-depleted HUVECs were analysed by ELISA to determine ADM concentrations. Interestingly, cortactin depletion resulted in a significant reduction of 39.8+/−3.1% of ADM concentrations in HUVEC supernatants (Fig. 4A). Furthermore, we analysed ADM concentrations in the plasma of cortactin KO and WT mice. ADM concentration was reduced by 66.5+/−7.3% in the plasma of KO mice (Fig. 4B). Real-time PCR data revealed a significant decrease by 51.8+/−7.3% in ADM mRNA production (Fig. 4C), which may in part explain the marked reduction of ADM plasma levels.

### ROCK1 inhibition and ADM administration reverse MLC phosphorylation and stress-fibre formation in cortactin-depleted endothelium

**Figure 5:**
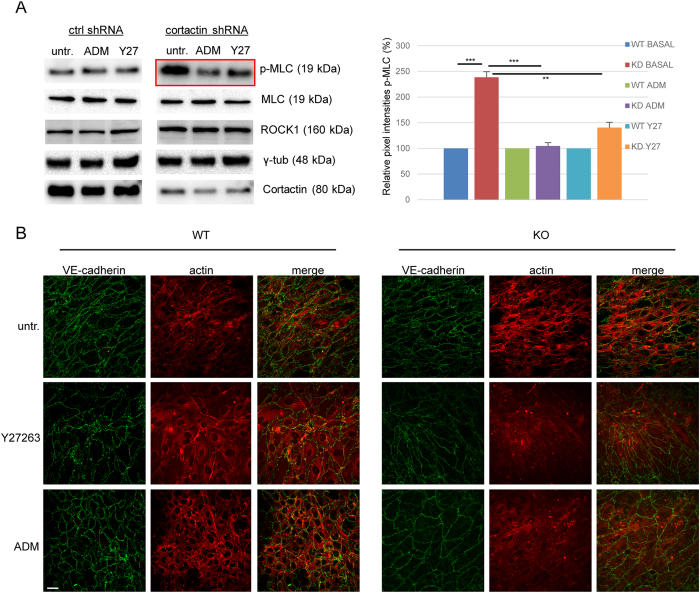
Adrenomedullin and ROCK1 inhibition counteract MLC phosphorylation and stress fibre formation. (**A**) Western blot experiments for the indicated proteins show that both ADM treatment (100 nM) and ROCK1 inhibition with Y27632 (Y27, 10 μM) for 1 h reverse the increase in MLC phosphorylation after cortactin depletion (red box), while total MLC levels are unaffected. γ-tubulin served as loading control. Graph shows quantification of p-MLC from 3 independent experiments (WT = ctrl shRNA; KD = cortactin shRNA). **p < 0.01; ***p < 0.001. (**B**) Immunofluorescence images of MLEC showing that both ROCK1 inhibition (Y27632) and ADM treatment counteract the increase in stress fibres (red) in the absence of cortactin (KO). VE-cadherin as cell contact staining is shown in green. Representative images from three independent experiments are shown. Bar = 20 μm.

ADM, similar to the effect of the specific EPAC (Rap1-GEF) activator 007, induces rapid activation of Rap1 within minutes^35^. Additionally, ADM pretreatment for 15 min is sufficient to prevent thrombin-induced MLC phosphorylation, stress fibre formation and permeability^40^. However, this has never been investigated before in the absence of cortactin. Thus, we analysed phosphorylation of MLC and ROCK1 expression by western blotting lysates from control and cortactin-depleted HMEC-1 treated with either ADM, the ROCK1 inhibitor Y27632 or vehicle. Incubation for 20 min yielded only a slight rescue effect (data not shown). We then increased the incubation time to 1 hour and found that ROCK1 protein levels were not affected by both treatments neither in control nor cortactin-depleted cells. Thus, neither ADM nor ROCK1 inhibition was able to counteract increased ROCK1 expression observed in cortactin-depleted cells (Fig. 5A). By contrast, both ADM administration and ROCK1 inhibition for 1 h reversed the increase in MLC phosphorylation in cortactin-depleted HMEC-1 with ADM having a stronger effect (Fig. 5A, red box). Quantification of pixel intensities of the p-MLC bands from 3 independent experiments revealed that ADM reversed the MLC phosphorylation levels in cortactin-depleted cells back to basal levels, while ROCK1 inhibition strongly reduced phosphorylation in these cells but not completely back to basal levels of control cells (Fig. 5A, graph). The effects of the treatment were statistically significant when compared to untreated WT cell but did not reach significance when the respective treated cells were compared. This finding confirms that ROCK1 protein is not only produced to a higher extent in the absence of cortactin, but that it is also more active. Additionally, our data imply that ADM is capable of inhibiting ROCK1 without affecting its production. Blots for cortactin, total MLC and γ-tubulin proved that neither silencing of cortactin nor expression of MLC or γ-tubulin were affected by ADM or ROCK inhibitor treatments (Fig. 5A). Additionally, we examined if ADM or Y27632 treatments could prevent the appearance of actin stress fibres in the absence of cortactin. To this end, we stained treated and untreated confluent WT or cortactin-KO MLEC for VE-Cadherin to visualise endothelial cell contacts and phalloidin to visualise actin fibres. As expected, cortactin-deficient MLEC showed increased actin fibres in comparison to control cells (Fig. 5B, compare Fig. 1A), while the VE-cadherin staining appeared in a similar distribution and intensity in both cell types, as observed previously^13^. Importantly, cortactin-deficient MLEC showed a marked reduction in actin stress fibres after both ADM administration and ROCK inhibition (Fig. 5B), suggesting that these treatments compensate for the effects of cortactin deficiency on stress fibre formation. Given that Rap1 activation occurs within a few minutes downstream of ADM^35^, Rap1 activation precedes the observed effects on ROCK1-mediated MLC phosphorylation and reduction of stress fibres and permeability.

### Adrenomedullin and ROCK-1 inhibition counteract increased vascular permeability without cortactin

**Figure 6:**
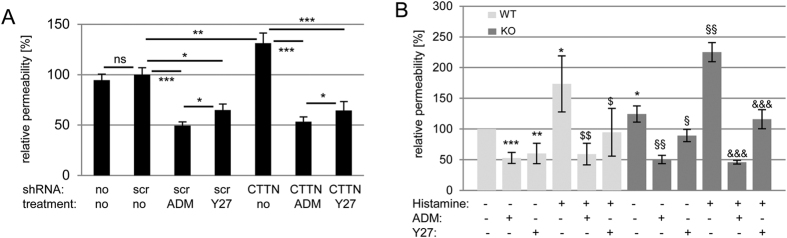
Adrenomedullin administration and ROCK1 inhibition rescue the increase in endothelial permeability provoked by the loss of cortactin. (**A**) HMEC-1 monolayers were cultured on 0.4 μm transwell filters until confluent. Fresh medium was added to the upper and lower chambers. Cells were untreated or treated for 1 h with 100 nM ADM or 10 μM Y-27632. Then, 150 kDa FITC-dextran was added to the upper chamber and incubated for 30 min. 100 μl were collected from the lower chamber and signal intensity was measured using a fluorometer. Data are mean +/−SDM of three independent experiments and represent relative permeability with control cells expressing scrambled (scr) shRNA set to 100%. *p < 0.05; **p < 0.01; ***p < 0.001. (**B**) Miles assays to determine vascular permeability in the skin were performed as described in Methods with the indicated treatments. WT: n = 8; KO: n = 5 from 2 independent experiments. Data are presented as relative permeability with the untreated WT control group set to 100%. Statistical analysis was performed by One-way ANOVA with Bonferroni’s post-hoc test. *p vs WT ctrl; ^$^p vs. WT + histamine; ^§^p vs. KO ctrl; ^&^p vs. KO + histamine; three symbols: p < 0.001, two symbols: p < 0.01; one symbol: p < 0.05.

Since ADM administration and ROCK1 inhibition significantly reduced MLC phosphorylation and actin stress fibres, we hypothesised that these mechanisms would be sufficient to reverse the increased permeability provoked by the absence of cortactin. To test this idea, we cultured control and cortactin-depleted HMEC-1 on 0.4 μm transwell filters to confluency and treated them with 100 nM ADM, 10 μM Y27632 or vehicle for 1 h before assessing paracellular flux using 150 kDa FITC-dextran. Infection of HMEC-1 with scrambled shRNA lentiviral particles did not affect the paracelular permeability of 150 kDa FITC-dextran when compared with uninfected HMEC-1 cells (Fig. 6A). Control cells treated with ADM or Y27632 showed reduced permeability as expected, with ADM being a more potent barrier enhancer. Cortactin-depleted cells showed an increase in permeability under basal conditions as previously reported^13^. Of note, the higher permeability of cortactin-depleted cells was counteracted by treatments with both ADM and Y27632 to levels comparable to control cells. ADM enhanced the barrier more potently than Y27632 suggesting that ADM has barrier-stabilising effects beyond ROCK inhibition. These data confirm the observations for actin stress fibres and MLC phosphorylation (Fig. 5) and verify ROCK1 and ADM as key regulators of endothelial permeability. To prove the physiological relevance of our findings, we performed Miles assays to determine vascular permeability in the skin of mice in the presence or absence of a permeability-inducing stimulus (histamine). Cortactin-deficient mice showed higher basal and induced permeability as expected. Both ADM and Y27632 reduced basal and induced permeability in WT mice, again with ADM showing a more potent effect (Fig. 6B). Importantly, Y27632 was able to significantly reduce the increased permeability under basal and induced conditions in cortactin-KO mice, whereas ADM administration completely reversed these increases to control levels. These data demonstarte that disturbed ADM secretion and downstream signaling is responsible for endothelial barrier dysfunction in cortactin-KO mice.

## Discussion

**Figure 7:**
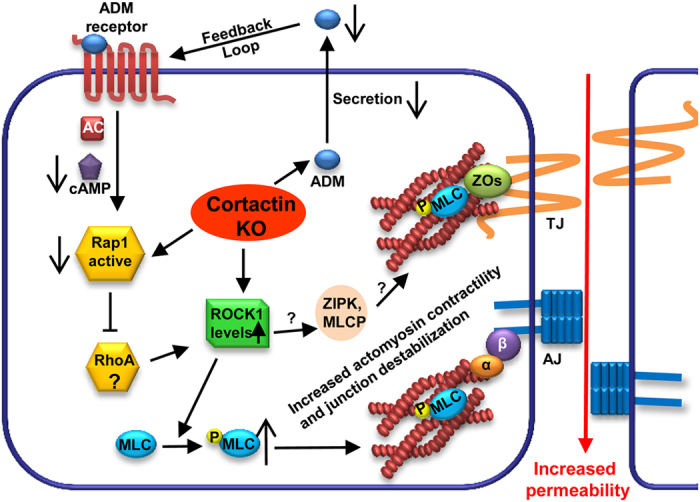
Consequences of cortactin depletion in endothelial cells. Loss of cortactin leads to decreased adrenomedullin secretion, reduced cAMP-dependent Rap1 activation, increased ROCK-1 protein levels, increased MLC phosphorylation and contractile stress-fibre formation and as a consequence increased endothelial permeability. If other ROCK1 targets such as zipper-interacting protein kinase (ZIPK) or myosin light chain phosphatase (MLCP) also contribute to barrier regulation in this system is not yet known (question marks).

Here, we analysed the actin cytoskeleton in cortactin-deficient endothelial cells and found for the first time that cortactin is involved in the regulation of actomyosin contractility. Cortactin deficiency increased ROCK1 protein levels and decreased ADM secretion accompanied by elevated MLC phosphorylation and actomyosin contractility causing increased endothelial permeability (Fig. 7).

The *in vitro* role of cortactin in important cellular processes that require actin remodelling and Arp2/3-complex function have been extensively studied^16^,^41^. However, analysis of cortactin-deficient fibroblasts revealed that cortactin is not essential for Arp2/3-dependent lamellipodia formation^42^, but may instead just tune its activity; for instance by stabilizing branches and/or antagonizing the activity of the branch disassembly factor coronin 1B^43^. Hitherto, cortactin has not yet been implicated in the regulation of stress fibres. In this study, we provide evidence that the endothelial phenotype of cortactin-deficient mice, as reported earlier, is caused by increased actomyosin contractility as a consequence of decreased ADM secretion leading to endothelial dysfunction as manifested by increased permeability.

There is strong evidence suggesting that cortactin is involved in the regulation of actin dynamics to promote cell functions such as adhesion and migration but also stabilisation of intercellular contacts^9^,^44^. We have recently shown that cortactin deficiency is associated with increased vascular permeability due to decreased activity of Rap1 13. Moreover, cortactin can contribute to S1P- or ATP-mediated stabilisation of endothelial junctions by translocating to cell contacts where it facilitates activation of Rac1, MLCK and actin remodelling^45^,^46^. On the other hand, ROCK1 has been suggested to be an important regulator of actomyosin contractility not only by controling MLCK and MLCP activities but also by directly phosphorylating MLC at serine-19 and threonine-18^47^,^48^,^49^,^50^,^51^. Both residues are known to contribute to myosin motor activity^52^. Since no antibodies are available that specifically recognize myosin phosphorylated on threonine-18, we analyzed MLC phosphorylation using antibodies that recognize p-serine19 and the dually phosphorylated form p-Thr18/p-ser19, respectively. Both antibodies detected a similar increase in phosphorylation status (compare Fig. 3A) suggesting that MLC is dually phosphorylated at thr-18 and ser-19 in endothelial cells lacking cortactin.

Rho A regulates the activity of myosin II and ROCK and is consequently responsible for intracellular tension^54^. It has been known for several years that RhoA and ROCK activation downstream of many permeability-increasing mediators such as thrombin or TNF-α contributes to increased permeability^23^. Inhibition of ROCK reduced baseline permeability in post-capillary venules *in vivo* and in different cultured microvascular endothelial cells^55^. The underlying mechanism is thought to be the prevention of contractile stress fibres that pull on cell-cell junctions^29^. Contractility per se induces stress fibre formation by further increasing the activity of RhoA/ROCK1 54. Moreover, formation of stress fibres after TNF-α-mediated activation of the RhoA/ROCK pathway increased permeability in HUVEC monolayers^56^. Since Rap1 is known to inhibit the RhoA/ROCK axis^25^, it seems logical to deduce that cortactin acts as a suppressor of this pathway by maintaining stable levels of active Rap1. MLCK is also involved in the formation of actin stress fibres and ROCK1 can directly phosphorylate and activate it to induce more contractile forces leading to increased permeability^47^. In addition, cortactin has been found to co-immunoprecipitate with MLCK suggesting a potential role for cortactin in the regulation of this protein^19^,^57^. However, MLCK levels were not increased in our endothelial models and ROCK1 inhibition was sufficient to prevent MLC phosphorylation, which suggested instead that cortactin controls ROCK1-mediated actomyosin contractility. We cannot formally rule out cortactin-specific effects on RhoA activity, but ROCK can also act independently of RhoA activation. For example, PKC activation in intestinal epithelial cells led to direct and RhoA-independent activation of myosin IIA and subsequent junction disassembly that could be ameliorated by inhibition of ROCK but not RhoA or MLCK^58^. Thus, it is tempting to speculate that cortactin deficiency contributes to the activation of signalling pathways directly triggering ROCK activation such as PKC. However, this hypothesis needs to be carefully evaluated in future studies.

Cortactin KO mice showed higher permeability under basal conditions compared to WT mice due to reduced levels of active Rap1 and this could be counteracted by a cAMP analogue that specifically activates the Rap1-GEF EPAC^13^. Thus, we hypothesized that endogenous inducers of cAMP synthesis involved in Rap1 activation and endothelial barrier regulation could be affected by the loss of cortactin. ADM is an endogenous peptide hormone known to enhance barrier stability in many ways, one of which being the elevation of cAMP levels for example in rabbit lung perfusates where it reduces vascular hyperpermeability^40^. ADM has also been suggested as a potential therapeutic molecule to counteract cardiovascular malfunctions^59^. Since ADM is produced by endothelial cells and activates adenylyl cyclase to produce cAMP, we tested if cortactin-deficiency led to disturbed ADM secretion. This idea is further supported by previous findings showing that cortactin controls secretion of the extracellular matrix protein fibronectin and metallo-proteases to regulate migration and invasion^60^,^61^. Indeed, blood plasma of cortactin-deficient mice as well as supernatants of cortactin-depleted human endothelial cells showed significantly reduced levels of secreted ADM. Real-time PCR also showed a decrease in ADM production. This, however, may not be sufficient to explain the marked reduction of ADM plasma levels. It is currently unknown how cortactin deficiency affects transcription, but it has been shown that cortactin can regulate transcription via RhoA^62^. Another explanation could be the altered G- to F-actin ratio since this can also affect transcription of various target genes via the serum response factor (SRF) pathway^63^. Future studies will have to establish the nature and specificity of ADM transcription regulation by cortactin. Of note, Western blots of MLEC cell lysates showed very little to no intracellular ADM (not shown), which is in agreement with a previous report showing that ADM is constitutively secreted and not stored in endothelial cells^64^. Thus, whether or not the reduced levels of ADM expressed in the absence of cortactin is also inefficiently secreted due to potential trafficking defects, as observed previously^65^,^66^, remains to be investigated.

Importantly, endothelial monolayers treated with ADM showed less MLC phosphorylation and stress fibre formation and reversed the increased permeability without cortactin *in vitro* and *in vivo*. In combination with the fact that ADM KO embryos die at midgestation due to vessel malformations and increased vascular permeability leading to excessive oedema formation manifested as extreme hydropsis fetalis^67^, it is reasonable to conclude that reduced ADM secretion and ADM-induced Rap1 activation are responsible for the endothelial barrier dysfunction in the absence of cortactin (Fig. 7). On the other hand, ADM also activates PKA via cAMP and PKA has also been shown to stabilise endothelial barrier functions^68^. Since ADM showed similar effects as the EPAC-specific cAMP analogue that does not activate PKA^13^, we assume that ADM/cAMP-dependent EPAC/Rap1 activation is the predominant barrier-stabilising pathway in this context. However, endothelial permeability is reduced to comparable levels in WT and cortactin-depleted cells by ADM whereas ROCK inhibition also shows a significant reduction albeit not to the same extent as ADM. These data suggest that ADM can act through other pathways additional to ROCK1 inhibition to stabilise endothelial barriers. If activation of PKA or other yet to be identified pathways trigger this additional barrier-stabilising effect remains to be unravelled.

In brain and lung tissue sections, we observed stronger ROCK1 staining in cortactin-deficient mice that is mostly restricted to blood vessels suggesting specific cortactin functions in endothelial cells compared to other cell types. Moreover, PECAM-1 protein levels also appeared to be upregulated in cells lacking cortactin. PECAM-1 has been reported to counteract ICAM-1-mediated tyrosine phosphorylation of several actin-binding proteins and to participate in the rearrangement of the actin cytoskeleton, thus maintaining the integrity of brain endothelium^69^. Moreover, it has been shown that PECAM-1 deficiency leads to increased permeability both *in vitro* and *in vivo*^70^. Together with our findings, these data suggest that PECAM-1 overexpression could counteract ROCK1-induced permeability in the absence of cortactin. It will be interesting to investigate whether loss of cortactin also affects the composition of interendothelial junctions to regulate endothelial barrier functions.

In conclusion, our data provide evidence that cortactin is essential for endothelial barrier integrity by controlling the molecular machinery regulating ADM secretion and actomyosin contractility. Since vascular hyperpermeability is a hallmark of many inflammatory disorders, it will be important to study if failure of cortactin-mediated control of actomyosin contractility could be a mechanism triggering the progression of such inflammations.

## Materials and Methods

### Antibodies and reagents

ADM, histamine, 150 kDa FITC-dextran and Y27632 were purchased from Sigma-Aldrich (Toluca, Mexico). The following antibodies have been used: monoclonal anti-cortactin (clone 4F11, 1:3000, Millipore, Naucalpan, Mexico); anti-γ-tubulin (clone GTU-88, 1:5000) and polyclonal anti-MLCK (SAB1300116, 1:500) (Sigma-Aldrich), monoclonal anti-PECAM-1 (clone MEC13.3., 1:400, BD, Franklin Lakes, NJ); polyclonal anti-ROCK1 (#4035, 1:1000), anti-MLC (#3672, 1:1000), anti-pMLC (#3671, Ser-19, 1:1000), anti-pMLC (#3674, Thr-18/Ser-19, 1:1000), anti-mDia1 (clone 51/mDia1, 1:1000), anti-ezrin (#3145, 1:1000), anti-p-ezrin (#3149, 1:1000), anti-cofilin (#3312S, 1:1000) and anti-p-cofilin (#3313P, 1:1000) (Cell Signaling, Danvers, MA), anti-VE-cadherin (C19, sc6458, 1:400) and species-specific peroxidase-labelled secondary antibodies (1:5000, Santa Cruz Biotechnologies, Santa Cruz, CA). Alexa-568-phalloidin (1:100, A12380; and species-specific Alexa-labelled secondary antibodies (1:2000) were from Invitrogen (Carlsbad, CA).

### Cell culture

Human microvascular endothelial cells (HMEC-1, passages 20–30 with recently confirmed phenotype and free of contaminations, generously provided by Dr. Isaura Meza) were cultivated as described^71^. Human umbilical vein endothelial cells (HUVEC) were isolated from discarded human umbilical cords (informed consent was obtained from all donors) as described and used between passage 3–5 72. Murine lung endothelial cells (MLEC) were isolated from C57Bl/6J WT mice and cortactin KO mice on a C57Bl/6J genetic background (at least 15 backcrosses) and cultivated as described^13^,^73^. Protocols to isolate HUVEC and MLEC have been approved by the bioethics committee and the institutional animal care and use committee of CINVESTAV (Mexico-City, Mexico) and all methods have been carried out in accordance to the approved guidelines.

### Generation of stable cortactin-depleted HMEC-1

Cortactin-depleted HMEC-1 cells were generated using a trans-lentiviral packaging kit according to the manufacturer’s instructions (Thermo Scientific, Waltham, MA). The vector pLKO.1 with a puromycin-resistance cassette (Addgene, Cambridge, MA, USA) was used to introduce the shRNA sequences (scrambled: CGGAGAAGTGGAGAAGCATAC and cortactin-directed: cortactin1: CACCAGGAGCATATCAACATA and cortactin2: AAGCTGAGGGAGAATGTCTTT). Cortactin1 showed better and consistent downregulation efficiencies of 80–90% and was used for the shown experiments. To generate viral particles, HEK293T cells were transfected at a confluency of 70% using serum-free DMEM containing 9 μg of each plasmid (packaging + shRNA) and 18 μg/ml polyethyleneimine (PEI). Virus containing media were collected 48 h and 72 h post-transfection. HMEC-1 cells were transduced with a MOI of 10 for 4 h at 37 °C and then incubated for 24 h with fresh medium. To select infected cells, medium was replaced with fresh medium containing 1.5 μg/ml puromycin.

### Transfection of HUVEC with siRNA

In contrast to HMEC-1, HUVEC are not stable for many passages so that we used a siRNA-based approach for cortactin depletion. HUVEC were transfected in passage three with the nucleofector device (Lonza, Basel, Switzerland) according to the manufacturer’s instructions, and using the cortactin-directed siRNAs cortactin 5 and 6 and a scrambled control (Qiagen, Hilden, Germany). Transfected cells were subjected to further analysis 48 h after transfection.

### qRT-PCR

Total RNA was isolated from WT and cortactin KO MLEC lysed in TRIzol (Invitrogen, Carlsbad, CA) according to the manufacturer’s instructions followed by phenol-chloroform extraction. cDNA was synthesized by reverse transcription using Superscript II and oligo(dT_12–18_) primers (Invitrogen). PCRs were carried out in a total volume of 10 μL, containing 5.0 μL 2x Power SYBR Green PCR Master Mix, 100 ng cDNA, and 0.15 μM of each primer in a StepOne^TM^ Real-Time PCR System (Applied Biosystems, Mexico-City, Mexico). Forward and reverse primers were located in different exons to prevent amplification of genomic DNA. Primer sequences were: ROCK-1-FW: ATGTGATACAGCGGTTGGAA and ROCK-1-RE: CGACCACCAGTCACATTCTC; cortactin-FW: AATTCGGTGTTCAGTCGGAG; cortactin-RE: TATCCATCCGATCCTTCTGC; ADM-FW: GTCGTGGGAAGAGGGAACTA; ADM-RE: TCTGATTGCTGGCTTGTAGG; GAPDH-FW: TGTTGCCATCAATGACCCCTT and GAPDH-RE: CTCCACGACGTACTCAGCG; U1-FW: CCATGATCACGAAGGTGGTTT. Primers were obtained from Uniparts (Mexico-City, Mexico). PCR conditions were: 95 °C for 10 min followed by 40 cycles of 95 °C for 15 s, 62 °C (57 °C for MLCK) for 12 s and 72 °C for 15 s. Amplicon sizes were verified by electrophoresis. Data from 4 independent cDNA preparations were analyzed using the ∆∆C_t_ method with β–actin as house-keeping gene as described^74^.

### Western blotting

Equal protein amounts of cell lysates were separated by polyacrylamide gel electrophoresis and transferred electrophoretically to PVDF membranes (Millipore). After incubation in TBS containing either 5% skim milk or 3% BSA (in case of blots for phosphorylated proteins) for 1 h to block unspecific binding, the membranes were probed with primary antibodies at 4 °C overnight, washed three times in TBS containing 0.05% Tween-20 for 10 min each and incubated with species-specific peroxidase-conjugated secondary antibodies for 1 h. Chemiluminescence signals were recorded on a ChemiDoc imaging device (Biorad, Mexico-City, Mexico). γ-Tubulin blots were performed as loading controls.

### G- to F-actin ratio

Determination of G/F-actin ratios have been performed using the G-actin/F-actin *in vivo* assay Biochem kit according to the manufacturer’s instructions (Cytoskeleton Inc., Denver, CO).

### Immunofluorescence microscopy

Cells or tissues were fixed in 100% ethanol at −20 °C for 20 min. After washing, cells were incubated for 1 h in PBS containing 2% normalised goat serum to block unspecific binding. Immunolabeling with primary antibodies was performed overnight at 4 °C followed by washing and incubation with species-specific fluorescently-labeled secondary antibodies and/or Alexa568-labeled phalloidin. Preparations were mounted in fluorescent mounting medium (DAKO Cytomation) and examined using a laser scanning confocal imaging system (FV-300, Olympus, Miami, FL). To reduce autofluorescence, lung tissue sections were incubated after fixation in 0.2% NaBH_4_ for 5 minutes and after secondary antibody incubation in 0.1% Sudan Black B diluted in 70% ethanol during 5 minutes at room temperature followed by thorough washing with water. Quantification of fluorescence intensities was performed using Image J software (NIH). Ten fields per image of three representative images out of at least three independent preparations were randomly selected and analysed. Pixel intensities of the WT conditions were set as 100% and the corresponding KO signals were expressed as percent of WT control. Additional central actin fibre density quantifications were performed using ImageJ as described^28^, with the modification that a line was drawn across cell centers (excluding cortical actin at cell contacts), across which the density ratio was determined.

### Detection of ADM concentrations

Blood was taken from 10–12 week old male C57Bl/6 WT or cortactin KO mice by puncture of the retro-orbital plexus and collected in centrifuge tubes containing 50 mM sodium citrate. Alternatively, supernatant of confluent monolayers of HUVEC were collected after three days of confluence. Samples were centrifuged for 10 min at 1200 rpm to remove all cells. Content of ADM in the plasma of WT and cortactin KO mice or cell culture supernatants was then determined using a colorimetric ELISA kit (Phoenix Pharmaceuticals, Karlsruhe, Germany) according to the manufacturer’s instructions.

### Permeability assay

Stable cortactin-depleted or scrambled HMEC-1 were seeded at a density of 5 × 10^4^ on 6.5-mm-diameter transwell filters (Corning) with 0.4 μm pore size, coated with Attachment Factor (Invitrogen) and cultured for 48 h before starting the assay. Cells were preincubated for 1 h with or without 100 nM ADM or 10 μM Y27632 before 0.25 mg/ml FITC-dextrane (150 kD) was added to the upper chamber. 30 min after administration, fluorescence in the lower chamber was measured using a spectrofluorimeter (Synergy-2; Bio-Tek). Confluence of cells was determined by immunofluorescence staining for VE-cadherin for each assay.

### *In vivo* determination of vascular permeability

Animal protocols have been approved by the institutional animal care and use committee of CINVESTAV (Mexico-City, Mexico) and all methods have been carried out in accordance to the approved guidelines. Modified Miles assays were performed using male 8–12-week-old C57Bl/8J WT and cortactin-KO mice on a C57Bl/8J WT background (at least 15 backcrosses). 10 min after injection of 1.5% Evan’s blue dye in PBS into the tail vein, 50 μl PBS or 100 ng histamine in 50 μl PBS with or without 1 μM ADM or 10 μM Y27632 were intradermally injected into the shaved back skin. Mice were sacrificed 30 min later and the skin patches of the sites of injections were excised and incubated in formamide for 5 days at room temperature. The extracted dye was then spectrophotometrically measured at 620 nm. Values are expressed as percent of the WT control.

### Statistical analysis

Data are presented as arithmetric mean with standard deviation of the mean (SDM). Statistical significance was assessed using Student’s t-test or one-way ANOVA including Bonferroni’s multiple comparison test where appropriate. A p-value of <0.05 was considered statistically significant.

## Additional Information

**How to cite this article**: García Ponce, A. *et al*. Loss of cortactin causes endothelial barrier dysfunction via disturbed adrenomedullin secretion and actomyosin contractility. *Sci. Rep*. **6**, 29003; doi: 10.1038/srep29003 (2016).
